# Self‐reported physical activity and gait in older adults without dementia: A longitudinal study

**DOI:** 10.1002/hsr2.70108

**Published:** 2024-11-06

**Authors:** Janina Krell‐Roesch, Jeremy A. Syrjanen, Tobias Moeller, Jelena Krafft, Bettina Barisch‐Fritz, Walter K. Kremers, Farwa Ali, David S. Knopman, Ronald C. Petersen, Thorsten Stein, Alexander Woll, Maria Vassilaki, Yonas E. Geda

**Affiliations:** ^1^ Institute of Sports and Sports Science Karlsruhe Institute of Technology Karlsruhe Germany; ^2^ Department of Quantitative Health Sciences Mayo Clinic Rochester Minnesota USA; ^3^ Department of Neurology Mayo Clinic Rochester Minnesota USA; ^4^ Department of Neurology and the Franke Barrow Global Neuroscience Education Center Barrow Neurological Institute Phoenix Arizona USA

**Keywords:** fall risk, gait, longitudinal, physical activity

## Abstract

**Background and Aims:**

Physical activity (PA) is associated with higher gait speed. We aimed to examine the associations between PA and change in spatial and temporal gait measures as well as fall risk in community‐dwelling individuals free of dementia.

**Methods:**

Longitudinal study among 4173 individuals aged ≥50 years (mean age 71 years; 2078 males; median follow‐up 4 years) enrolled in the Mayo Clinic Study of Aging. Self‐reported late‐life PA was used to calculate overall PA and moderate‐vigorous PA (MVPA) scores. Gait was assessed using GAITRite® and Zeno™ systems. Incident falls information was based on diagnostic codes retrieved from medical records. We ran linear mixed effects models to examine associations between z‐scored PA variables and longitudinal gait parameters, adjusted for age, sex, education, body mass index (BMI), medical comorbidities, and including interactions between PA and time since baseline. In secondary analyses, we calculated Cox Proportional hazard models with age as time scale predicting incident falls by PA, adjusting for sex, education, BMI, medical comorbidities, and falls history.

**Results:**

At baseline, higher PA was associated with higher velocity (overall PA: estimate 2.9935; MVPA: 2.2961; *p* < 0.001), higher cadence (overall PA: 1.0665; MVPA: 0.9073; *p* < 0.001), greater stride length (overall PA: 2.0805; MVPA: 1.4726; *p* < 0.001), shorter double support time (overall PA: −0.0257; MVPA: −0.0205; *p* < 0.001), and lower stance time variability (overall PA: −0.0204, *p* < 0.001; MVPA: −0.0152; *p* = 0.006). Overall PA was longitudinally associated with less decline in cadence, and MVPA with less increase in intraindividual stance time variability. Overall PA (Hazard ratio 0.892, 95% confidence interval 0.828–0.961, *p* = 0.003) and MVPA (HR 0.901; 95% CI 0.835–0.973, *p* = 0.008) were associated with a decreased risk of incident falls.

**Conclusion:**

Late‐life PA was associated with favorable gait outcomes and decreased risk of incident falls. Thus, late‐life PA may help to maintain gait performance and decrease fall risk in old age.

## Key points

- We examined the associations between physical activity in late‐life and change in spatial and temporal gait measures as well as fall risk in 4173 community‐dwelling persons aged ≥50 years and free of dementia enrolled in the Mayo Clinic Study of Aging.
- Higher overall physical activity and moderate‐vigorous physical activity are associated with higher velocity, higher cadence, greater stride length, shorter double support time, and lower stance time variability.
- Higher overall physical activity is longitudinally associated with less decline in cadence, higher moderate‐vigorous physical activity with less increase in intraindividual stance time variability, and both with a decreased risk of incident falls.

## 1 INTRODUCTION

Gait performance declines with increasing age.^1^, ^2^ Gait in older adults is characterized by various alterations as compared to gait in younger individuals, including but not limited to reduced speed, shorter stride length, and increased gait variability,^3^, ^4^ which are in turn associated with an increased risk of falling.^5^ Decreases in gait performance are also predictive of cognitive decline,^6^ and have been linked to neuropathological changes of Alzheimer's disease.^7^, ^8^, ^9^

Physical activity is associated with slower rate of motor decline,^10^ and better motor performance outcomes including gait speed in older persons.^11^, ^12^ In addition, physical activity interventions may have a favorable impact on gait performance.^13^ Research has shown that particularly higher physical activity intensities are associated with better gait,^14^ and that physical activity interventions may be associated with gait even in dementia patients.^15^

Few studies have examined associations between physical activity and different gait parameters in older adults. For example, a cross‐sectional study among 55 older adults showed that sedentary as compared to physically active individuals had shorter step lengths and slower step velocities.^16^ Similarly, a recent cross‐sectional study in a small sample of 16 older adults showed that physical activity level was associated with higher gait quality,^3^ and another reported associations between higher physical activity frequency with better gait performance, i.e. gait speed and stride length.^17^ The longitudinal associations between physical activity and gait in older adults have been examined by only few studies. For example, one study showed that moderate‐vigorous physical activity (MVPA) was associated with faster gait speed over time in adults aged ≤ 70 years, and that low‐intensity physical activity was associated with faster gait speed in adults aged ≥ 70 years.^18^ Another study reported that declines in walking speed were longitudinally associated with decreased physical activity, but physical activity was not associated with later walking speed changes.^19^ Similarly, another study also provided evidence of an association between gait speed with longitudinal change in physical activity.^20^ However, to date, little is known about the longitudinal associations between physical activity as predictor, and various spatial and temporal gait measures as outcomes of interest in large, population‐based samples of older adults.

Therefore, we examined whether physical activity was associated with change of multiple gait parameters in community‐dwelling persons free of dementia. In secondary analyses, we examined the associations between physical activity and risk of incident falls.

## 2 METHODS

### 2.1 Study setting and design

The study was conducted in the setting of the population‐based Mayo Clinic Study of Aging in Olmsted County, Minnesota, USA.^21^ We included 4173 individuals aged ≥ 50 years with available information on physical activity within 12 months of baseline assessment, various spatial and temporal gait parameters, information about falls from medical charts, and covariates. Participants were followed forward in time for a median of 4.09 years for the gait outcomes, and for the outcome of incident falls, median follow‐up time was 9.45 years (29 participants did not have follow‐up for incident falls and were excluded from those models). The Mayo Clinic Study of Aging protocols have been approved by the institutional review boards of the Mayo Clinic and Olmsted Medical Center in Rochester, MN, USA. Participants provided written informed consent. In the case of participants with cognitive impairment sufficient to interfere with capacity, assent was obtained from a legally authorized representative.

### 2.2 Measurement of physical activity (predictor variables)

Physical activity was measured using a self‐reported questionnaire.^22^ The questionnaire was derived from two validated instruments, i.e., the 1985 National Health Interview Survey and the Minnesota Heart Survey intensity codes.^23^, ^24^ The questionnaire inquired about engagement in physical activity and exercise in late‐life (i.e., within 12 months of baseline assessment) and distinguished between three intensity levels by providing examples for each level: (1) light physical activity (such as laundry, vacuuming, making beds or dusting); (2) moderate physical activity (such as scrubbing floors, washing windows, gardening or raking leaves); (3) heavy physical activity (such as carrying heavy objects, heavy digging, pushing a mower or hard manual labor); (4) light physical exercise (such as leisurely walking or slow dancing); (5) moderate physical exercise (such as hiking or swimming); and (6) vigorous physical exercise (such as jogging or playing tennis singles). Participants were asked to provide information about the frequency at which they carried out these activities: ≤1 time per month, 2–3 times per month, 1–2 times per week, 3–4 times per week, 5–6 times per week, and daily. For the purpose of this study, we first assigned the following metabolic equivalent (MET) to the intensity categories based on published MET values for different physical activities^25^: light physical activity (2.5 MET), moderate physical activity (4.0 MET), heavy physical activity (6.5 MET), light physical exercise (3.0 MET), moderate physical exercise (5.5 MET), and vigorous physical exercise (8.0 MET). We then calculated two scores: (1) a composite overall physical activity score by taking the MET for light, moderate, and heavy/vigorous physical activities and exercise, respectively, multiplying them with days per week at which the corresponding activity was carried out, and then adding them up to get an overall score; and (2) a MVPA score by adding the MET multiplied by days per week for moderate and vigorous physical exercise. Both scores were z‐ scored for use in the models; and a higher score reflects a higher level of physical activity similar to our previous publications.^26^, ^27^ The questionnaire to assess physical activity and exercise in the Mayo Clinic Study of Aging has moderate to good internal consistency, and test‐retest correlation coefficients of 0.33 for vigorous and 0.50 for moderate physical activity.^22^

### 2.3 Measurement of gait parameters (outcome variables)

Participants were instructed to walk at self‐selected speed down a 10‐m long GAITRite® (CIR Systems Inc., Franklin, NJ) or Zeno™ (ProtoKinetics LLC, Havertown, PA) pressure sensitivity walkway. Foot‐fall pressure data from the mat were processed using the Proto Kinetics Movement Analysis Software, which provides a wide range of gait variables for each foot separately and combined.^28^ We included the following spatial and temporal parameters in our analyses: (1) velocity: refers to walking speed in centimeter/second, product of cadence and step length; (2) cadence: refers to the number of steps per minute; (3) stride length: refers to the anterior‐posterior distance between the heels of two successive footprints made by the same foot (left to left, right to right), one stride or gait cycle consists of two steps (e.g., right step followed by left step); (4) double support time: refers to the period when both feet are touching the ground at the same time; double support time is the total duration of two periods of double support within the gait cycle; (5) intraindividual variability in stride length; and (6) intraindividual variability in stance time: refers to the duration from the initial to the last contact of a single footfall, the stance phase is the weight bearing part of each gait cycle starting with heel contact and ending with toe off of the same foot.^28^ Double support time as well as stride length standard deviation, and stance time standard deviation were all log transformed (base *e*) to improve model fits.

### 2.4 Measurement of incident falls (outcome variables)

The Rochester Epidemiology Project^29^, ^30^, ^31^ medical records linkage system was used to retrieve available diagnostic codes for falls from each participant's medical records (please refer to supplementary Material S1 for an overview of codes). Of note, these codes were developed as a broad capture screening mechanism, and we considered many fall diagnosis codes. We only considered incident falls as an outcome of interest in secondary analyses.

### 2.5 Covariates

We considered potential confounders, such as, age, sex, and education, as well as body mass index (body weight in kilograms/body height in meter squared) and medical comorbidity through the weighted Charlson Index.^32^ In the secondary analyses on the outcome of incident falls risk, we also included prior falls (i.e., prior to study baseline) as an additional confounder.

### 2.6 Statistical analysis

We ran linear mixed effects models with random subject‐specific intercepts and slopes (for years since baseline) to examine the association between late‐life physical activity and change in gait parameters over time; separate models were built for each gait parameter. In our models, the two physical activity variables (i.e., overall physical activity and MVPA scores), measured at baseline, were the independent variables (predictors), and longitudinal gait variables were the dependent variables (outcomes). All models included physical activity (z‐scored), sex, time in years from baseline, age at baseline, education years, body mass index at baseline, Charlson comorbidity index at baseline, and the interactions between physical activity and time in years from baseline. This model formulation allowed us to assess baseline associations between physical activity and gait variables, yearly trajectories of gait variables, and how physical activity might modify these trajectories. We conducted the analyses separately for overall physical activity and MVPA. In secondary analyses, we calculated Cox Proportional hazard models with age as the time scale, to predict incident falls (outcomes) from physical activity variables (predictors), adjusting for sex, education, body mass index, medical comorbidities, and history of falling. Some gait data appeared to have questionable validity likely due to an error during the walk. To mitigate this, we removed any that had an extreme outlier for any of the gait variables, which we defined as having a value less than Q1‐3*IQR or greater than Q3 + 3*IQR where Q1 is quartile 1, Q3 is quartile 3, and IQR is interquartile range. All statistical analyses were done using the conventional two‐tailed alpha level of 0.05 and performed with SAS 9.4 (SAS Institute, Inc; Cary, NC) and R version 4.2.2 (R Foundation for Statistical Computing; Vienna, Austria).

## 3 RESULTS

The characteristics of the study sample are summarized in Table 1. We included 4173 individuals aged ≥ 50 years (mean [SD] age 70.80 [9.95] years; 2078 males; 3763 cognitively unimpaired, 410 with mild cognitive impairment).

**Table 1:** Participant characteristics at study baseline.

Variable	*N* = 4173 Mean (SD)
Age in years	70.80 (9.95)
Male sex, *N* (%)	2078 (49.8)
Education in years	14.61 (2.61)
Body mass index	28.81 (5.68)
Charlson comorbidity index	3.03 (3.11)
Cognitive status	
Cognitively unimpaired, *N* (%)	3763 (90.2)
Mild cognitive impairment, *N* (%)	410 (9.8)
Late‐life overall PA score	55.01 (33.22)
Late‐life MVPA score	17.08 (18.64)
Velocity (cm/sec)	113.43 (21.70)
Cadence (steps/min)	107.27 (10.04)
Stride length (cm)	126.03 (19.76)^{69}^
Double support time (sec)	0.34 (0.07)^{69}^
Stride length (cm) variability	4.03 (1.74)^{69}^
Stance time (sec) variability	0.03 (0.02)^{69}^
Follow‐up in years	4.76 (4.06)

*Note*: Data presented as mean (SD) unless indicated otherwise. Stride length variability, SD of stride length; Stance time variability, SD of stance time; ^{N}^, indicates number of persons with missing data.

Abbreviations: cm, centimeter; min, minute; overall PA and MVPA scores, higher scores indicate higher overall and moderate‐vigorous physical activity, respectively; sec, second; SD, standard deviation.

At baseline, higher overall physical activity was associated with higher velocity (estimate 2.9935, *p* < 0.001), higher cadence (1.0665, *p* < 0.001), greater stride length (2.0805, *p* < 0.001), shorter double support time (−0.0257, *p* < 0.001), and lower stance time variability (−0.0204, *p* < 0.001). Similarly, higher MVPA was associated with higher velocity (2.2961, *p* < 0.001), higher cadence (0.9073, *p* < 0.001), greater stride length (1.4726, *p* < 0.001), shorter double support time (−0.0205, *p* < 0.001), and lower stance time variability (−0.0152, *p* = 0.006) at baseline.

Over time, participants with average levels of overall physical activity and MVPA, respectively, had annual decreases in velocity (overall physical activity: −1.6586, *p* < 0.001; MVPA: −1.6580, *p* < 0.001), cadence (overall physical activity: −0.1950, *p* < 0.001; MVPA: −0.1939, *p* < 0.001), stride length (overall physical activity: −1.6923, *p* < 0.001; MVPA: −1.6925, *p* < 0.001), and annual increases in double support time (overall physical activity: 0.0107, *p* < 0.001; MVPA: 0.0107, *p* < 0.001), stride length variability (overall physical activity: 0.0193, *p* < 0.001; MVPA: 0.0193, *p* < 0.001), and stance time variability (overall physical activity: 0.0126, *p* < 0.001; MVPA: 0.0126, *p* < 0.001), on average.

Higher levels of overall physical activity (i.e., one standard deviation above the mean) were longitudinally associated with less decline in cadence (−0.1950 + 0.0491 = −0.1459, interaction *p* = 0.02), and MVPA with less increase in intraindividual stance time variability (0.0126–0.0023 = 0.0103, interaction *p* = 0.03). Please refer to Table 2 for the full set of linear mixed effects models.

**Table 2:** Linear mixed effects models on late‐life physical activity and longitudinal gait parameters.

R2	Dependent variable	Term	Estimate	95% CI lower	95% CI upper	*p*
0.369	Velocity	Age	−1.0650	−1.1209	−1.0090	<0.001
		Male sex	3.8537	2.8403	4.8670	<0.001
		Education	0.7571	0.5619	0.9523	<0.001
		BMI	−0.9228	−1.0151	−0.8306	<0.001
		Comorbidities	−1.0290	−1.2053	−0.8527	<0.001
		Overall PA	2.9935	2.4560	3.5310	<0.001
		Time	−1.6586	−1.7425	−1.5747	<0.001
		Overall PA x time	0.0187	−0.0655	0.1030	0.66
0.366	Velocity	Age	−1.0756	−1.1318	−1.0194	<0.001
		Male sex	4.2135	3.1993	5.2277	<0.001
		Education	0.7038	0.5067	0.9009	<0.001
		BMI	−0.9537	−1.0463	−0.8612	<0.001
		Comorbidities	−1.0792	−1.2560	−0.9024	<0.001
		MVPA	2.2961	1.7577	2.8345	<0.001
		Time	−1.6580	−1.7418	−1.5741	<0.001
		MVPA x time	0.0350	−0.0476	0.1176	0.41
0.180	Cadence	Age	−0.1702	−0.1988	−0.1416	<0.001
		Male sex	−7.1683	−7.6862	−6.6505	<0.001
		Education	0.0934	−0.0063	0.1932	0.07
		BMI	−0.1572	−0.2043	−0.1101	<0.001
		Comorbidities	−0.2458	−0.3359	−0.1557	<0.001
		Overall PA	1.0665	0.7932	1.3397	<0.001
		Time	−0.1950	−0.2358	−0.1541	<0.001
		Overall PA x time	0.0491	0.0081	0.0900	0.02
0.178	Cadence	Age	−0.1726	−0.2012	−0.1440	<0.001
		Male sex	−7.0435	−7.5601	−6.5270	<0.001
		Education	0.0706	−0.0298	0.1710	0.17
		BMI	−0.1657	−0.2129	−0.1186	<0.001
		Comorbidities	−0.2622	−0.3523	−0.1722	<0.001
		MVPA	0.9073	0.6347	1.1799	<0.001
		Time	−0.1939	−0.2347	−0.1530	<0.001
		MVPA x time	0.0385	−0.0017	0.0787	0.06
0.449	Stride length	Age	−0.9764	−1.0249	−0.9279	<0.001
		Male sex	13.0362	12.1575	13.9148	<0.001
		Education	0.7370	0.5680	0.9059	<0.001
		BMI	−0.8319	−0.9119	−0.7519	<0.001
		Comorbidities	−0.9281	−1.0803	−0.7760	<0.001
		Overall PA	2.0805	1.6216	2.5394	<0.001
		Time	−1.6923	−1.7615	−1.6230	<0.001
		Overall PA x time	0.0030	−0.0667	0.0727	0.93
0.447	Stride length	Age	−0.9859	−1.0345	−0.9372	<0.001
		Male sex	13.2908	12.4124	14.1691	<0.001
		Education	0.7043	0.5339	0.8748	<0.001
		BMI	−0.8578	−0.9379	−0.7777	<0.001
		Comorbidities	−0.9657	−1.1181	−0.8133	<0.001
		MVPA	1.4726	1.0134	1.9318	<0.001
		Time	−1.6925	−1.7617	−1.6233	<0.001
		MVPA x time	0.0308	−0.0376	0.0992	0.38
0.287	Double support time	Age	0.0057	0.0052	0.0063	<0.001
		Male sex	0.0494	0.0399	0.0590	<0.001
		Education	−0.0030	−0.0049	−0.0012	0.001
		BMI	0.0145	0.0136	0.0153	<0.001
		Comorbidities	0.0064	0.0048	0.0081	<0.001
		Overall PA	−0.0257	−0.0308	−0.0206	<0.001
		Time	0.0107	0.0098	0.0116	<0.001
		Overall PA x time	−0.0001	−0.0009	0.0008	0.89
0.284	Double support time	Age	0.0058	0.0053	0.0064	<0.001
		Male sex	0.0465	0.0370	0.0560	<0.001
		Education	−0.0026	−0.0044	−0.0007	0.006
		BMI	0.0147	0.0138	0.0156	<0.001
		Comorbidities	0.0068	0.0052	0.0085	<0.001
		MVPA	−0.0205	−0.0256	−0.0154	<0.001
		Time	0.0107	0.0098	0.0116	<0.001
		MVPA x time	−0.0002	−0.0011	0.0006	0.57
0.064	Stride length SD	Age	0.0074	0.0065	0.0084	<0.001
		Male sex	0.0633	0.0469	0.0796	<0.001
		Education	0.0006	−0.0025	0.0038	0.70
		BMI	−0.0013	−0.0029	0.0002	0.09
		Comorbidities	0.0074	0.0044	0.0104	<0.001
		Overall PA	−0.0088	−0.0192	0.0016	0.10
		Time	0.0193	0.0174	0.0213	<0.001
		Overall PA x time	−0.0003	−0.0022	0.0016	0.78
0.064	Stride length SD	Age	0.0074	0.0065	0.0084	<0.001
		Male sex	0.0622	0.0459	0.0785	<0.001
		Education	0.0008	−0.0023	0.0040	0.61
		BMI	−0.0013	−0.0028	0.0002	0.10
		Comorbidities	0.0076	0.0046	0.0105	<0.001
		MVPA	−0.0080	−0.0184	0.0023	0.13
		Time	0.0193	0.0174	0.0213	<0.001
		MVPA x time	−0.0001	−0.0020	0.0018	0.93
0.115	Stance time SD	Age	0.0128	0.0118	0.0138	<0.001
		Male sex	−0.0197	−0.0377	−0.0017	0.03
		Education	−0.0069	−0.0104	−0.0034	<0.001
		BMI	0.0037	0.0021	0.0054	<0.001
		Comorbidities	0.0110	0.0078	0.0143	<0.001
		Overall PA	−0.0204	−0.0312	−0.0096	<0.001
		Time	0.0126	0.0105	0.0147	<0.001
		Overall PA x time	−0.0016	−0.0037	0.0005	0.15
0.115	Stance time SD	Age	0.0128	0.0118	0.0138	<0.001
		Male sex	−0.0224	−0.0403	−0.0044	0.01
		Education	−0.0064	−0.0099	−0.0029	<0.001
		BMI	0.0039	0.0023	0.0056	<0.001
		Comorbidities	0.0114	0.0082	0.0146	<0.001
		MVPA	−0.0152	−0.0260	−0.0044	0.006
		Time	0.0126	0.0105	0.0148	<0.001
		MVPA x time	−0.0023	−0.0044	−0.0002	0.03

*Note*: 95% CI, 95% confidence interval. Late‐life overall PA and MVPA were z‐scored and from baseline. Double support time, stride length standard deviation, and stance time standard deviation were all log transformed (base *e*).

Abbreviations: age, BMI and comorbidities (Charlson index) at baseline were entered into the models; BMI, body mass index; MVPA, moderate‐vigorous intensity physical activity; PA, physical activity; SD, standard deviation.

In secondary analyses, both overall physical activity (Hazard ratio 0.892, 95% confidence interval 0.828–0.961, *p* = 0.003) and MVPA (HR 0.901; 95% CI 0.835–0.973, *p* = 0.008) were associated with a decreased risk of incident falls.

## 4 DISCUSSION

Self‐reported late‐life physical activity, be it overall physical activity or MVPA, in community‐dwelling older adults free of dementia was associated with favorable spatial and temporal gait outcomes, and lower intraindividual variability of gait parameters at baseline. In addition, higher late‐life overall physical activity was associated with less decline in cadence, and higher late‐life MVPA with less increase in intraindividual variability of stance time. This observation highlights the importance of engaging in physical activity to maintain gait performance in old age.

Our study is in line with prior research that also reported associations between physical activity engagement and gait performance in older adults. For example, a study among 55 older adults showed that sedentary individuals had shorter step lengths and slower step velocities than physically active ones.^16^ A recent study in a small sample of 16 older adults also showed that physical activity level was associated with higher gait quality.^3^ A study among 608 older adults from the Rush Memory and Aging Project reported associations between accelerometer‐assessed physical activity with gait assessed through a body‐worn sensor during three tests (i.e., 32‐foot walk, Timed Up and Go, and 20‐second period of standing with closed eyes).^33^ Another study in which physical activity was also assessed objectively through accelerometry revealed associations between higher MVPA levels and lower gait variability.^34^ Only few longitudinal studies have been conducted on the associations between physical activity and gait in older adults, mainly focusing on gait speed. For example, a prospective study among persons aged 65 years and older with a follow‐up time of 4 years showed that physical inactivity was associated with an increased risk of developing incident slow gait.^35^ A study derived from the English Longitudinal Study of Ageing reported that MVPA was associated with faster gait speed over time in adults aged ≤ 70 years, and that low‐intensity physical activity was associated with faster gait speed in adults aged ≥ 70 years.^18^ Another study over a 9‐year follow‐up among 2876 older adults showed that declining walking speed was longitudinally associated with a decreased engagement in physical activity, but alterations in physical activity level were not associated with later walking speed changes.^19^ A Norwegian study in a large sample of older adults showed that gait speed was associated with daily step count, and that various gait parameters including gait speed, cadence, step length and time were associated with higher intensity physical activity and overall physical activity. In longitudinal analyses, only gait speed was associated with change in physical activity after 12 months follow‐up.^20^ Finally, a study from Japan in 782 older adults showed that faster gait speed but not physical activity was associated with decreased risk of incident disability, but that physical activity was a mediating factor between gait speed and mortality.^36^

However, as mentioned above, most studies are limited by small sample sizes and mainly focused on gait/walking speed. Thus, our study expands on the existing body of research by providing evidence of an association between overall physical activity as well as MVPA, with various spatial and temporal gait measures in a large, population‐based sample of older adults. Furthermore, we not only investigated cross‐sectional but also longitudinal associations between physical activity as predictor, and changes in gait parameters as outcomes. We were able to show that overall physical activity was associated with less decline in cadence, and MVPA with less increase in intraindividual variability of stance time. This finding may have implications for clinical practice and health counseling, and underlines the importance of engaging in physical activity in old age for preserving gait performance. Importantly, not only engaging in higher levels of MVPA, but also overall physical activity level was associated with better gait outcomes, which may be more relevant to older adults as many do not have the capacity to engage in moderate‐vigorous activities.

In secondary analyses, we observed that self‐reported engagement in physical activity in late‐life was also related to a decreased risk of incident falls. This has been reported for other population‐based samples in the past,^37^, ^38^, ^39^, ^40^ albeit conflicting findings have also been published,^41^ and further emphasizes the significance of engaging in physical activity for potentially decreasing fall risk in old age.

Strengths of our study are the rigorous assessment of different spatial and temporal gait variables using a well‐known, computerized and automated system, and the large sample of 4173 community‐dwelling persons aged ≥ 50 years. Limitations relate to the use of a self‐reported questionnaire to assess physical activity which may have led to recall bias. However, our team has published several manuscripts on physical activity data derived from this questionnaire in the past, and we have previously reported that the questionnaire has moderate to good internal consistency, and test‐retest correlation coefficients of 0.33 for vigorous and 0.50 for moderate physical activity.^22^ Furthermore, despite its longitudinal design, reverse causality may be a possible explanation of our findings (i.e., participants who develop gait problems over time are less likely to engage in physical activity in late‐life). In addition, more adjustments may be needed in future analyses (e.g., emotional health), and the gait parameters we report here are interrelated; thus, separate analyses models were developed for each gait characteristic. Furthermore, our sample is relatively highly educated and 98% of study participants are White, but data from Olmsted County are generalizable to the U.S. population of Minnesota and the Upper Midwest.^42^ More research is needed to examine the associations between objectively‐assessed physical activity and longitudinal changes in various gait parameters and fall risk over time, particularly in diverse populations.

## 5 CONCLUSION

We observed that late‐life physical activity is associated with favorable spatial and temporal gait outcomes. Higher levels of overall physical activity were longitudinally associated with less decline in cadence, and MVPA with less increase in intraindividual stance time variability. In addition, physical activity was also associated with a decreased risk of incident falls. This underlines the importance of engaging in physical activity to potentially maintain gait performance and decrease fall risk in community‐dwelling older adults.

## AUTHOR CONTRIBUTIONS

**Janina Krell‐Roesch**: Conceptualization; formal analysis; investigation; methodology; writing—original draft. **Jeremy A. Syrjanen**: Formal analysis; methodology; writing—review and editing. **Tobias Moeller**: Writing—review and editing. **Jelena Krafft**: Writing—review and editing. **Bettina Barisch‐Fritz**: Writing—review and editing. **Walter K. Kremers**: Formal analysis; methodology; supervision; writing—review and editing. **Farwa Ali**: Writing—review and editing. **David S. Knopman**: Funding acquisition; resources; supervision; writing—review and editing. **Ronald C. Petersen**: Funding acquisition; project administration; resources; writing—review and editing. **Thorsten Stein**: Writing—review and editing. **Alexander Woll**: Writing—review and editing. **Maria Vassilaki**: Conceptualization; investigation; methodology; supervision; writing—review and editing. **Yonas E. Geda**: Conceptualization; funding acquisition; methodology; project administration; supervision; writing—review and editing.

## CONFLICT OF INTEREST STATEMENT

WKK receives research funding from NIH, Astra Zeneca, Biogen, and Roche. DSK serves on a data safety monitoring board for the Dominantly Inherited Alzheimer Network TREATMENT UNIT (DIAN‐TU) study and was an investigator in clinical trials sponsored by Biogen, Lilly Pharmaceuticals, and the University of Southern California. RCP has consulted for Roche, Inc.; Genentech, Inc.; Eli Lilly, Inc.; Nestle, Inc. and Eisai, Inc.; a DSMB for Genentech, Inc. and receives royalties from Oxford University Press for Mild Cognitive Impairment and from UpToDate. His research funding is from NIH/NIA. MV consulted for F. Hoffmann‐La Roche Ltd, unrelated to this manuscript; she currently receives research funding from NIH and has equity ownership in Amgen, Johnson and Johnson, Medtronic, and Merck. YEG receives funding from Roche, served on the Lundbeck advisory board, and receives research funding from the NIH. The other authors report no disclosures.

## ETHICS STATEMENT

The Mayo Clinic Study of Aging protocols have been approved by the institutional review boards of the Mayo Clinic and Olmsted Medical Center in Rochester, MN, USA. Participants provided written informed consent. In the case of participants with cognitive impairment sufficient to interfere with capacity, assent was obtained from a legally authorized representative.

## TRANSPARENCY STATEMENT

The lead author Janina Krell‐Roesch affirms that this manuscript is an honest, accurate, and transparent account of the study being reported; that no important aspects of the study have been omitted; and that any discrepancies from the study as planned (and, if relevant, registered) have been explained.

## Supporting information


**Supplementary Material S1**. Codelist used for classification of falls for secondary analyses (those in red ink [i.e., repeated falls, history of falling] were only used for determining prior falls [confounding variable]).

## Data Availability

The data from the Mayo Clinic Study of Aging used in this study is available to qualified researchers upon reasonable request.
