# Supplementary material for: Self‐reported physical activity and gait in older adults without dementia: A longitudinal study
Source: Health Sci Rep. 2024 Nov 6;7(11):e70108. doi: 10.1002/hsr2.70108 (PMC11539020; doi:10.1002/hsr2.70108)
Supplement: Supplementary file 1 — Supplementary Material S1. Codelist used for classification of falls for secondary analyses (those in red ink [i.e., repeated falls, history of falling] were only used for determining prior falls [confounding variable]). [file HSR2-7-e70108-s001.docx]

**Supplementary Material S1**

ICD-10: W00.0XXA; W00.0XXD; W00.0XXS; W00.1XXA; W00.1XXD; W00.1XXS; W00.2XXA; W00.2XXD; W00.2XXS; W00.9XXA; W00.9XXD; W00.9XXS; W01.0XXA; W01.0XXD; W01.0XXS; W01.10XA; W01.10XD; W01.10XS; W01.110A; W01.110D; W01.110S; W01.111A; W01.111D; W01.111S; W01.118A; W01.118D; W01.118S; W01.119A; W01.119D; W01.119S; W01.190A; W01.190D; W01.190S; W01.198A; W01.198D; W01.198S; W03.XXXA; W03.XXXD; W03.XXXS; W04.XXXA; W04.XXXD; W04.XXXS; W05.0XXA; W05.0XXD; W05.0XXS; W05.1XXA; W05.1XXD; W05.1XXS; W05.2XXA; W05.2XXD; W05.2XXS; W06.XXXA; W06.XXXD; W06.XXXS; W07.XXXA; W07.XXXD; W07.XXXS; W08.XXXA; W08.XXXD; W08.XXXS; W09.0XXA; W09.0XXD; W09.0XXS; W09.1XXA; W09.1XXD; W09.1XXS; W09.2XXA; W09.2XXD; W09.2XXS; W09.8XXA; W09.8XXD; W09.8XXS; W10.0XXA; W10.0XXD; W10.0XXS; W10.1XXA; W10.1XXD; W10.1XXS; W10.2XXA; W10.2XXD; W10.2XXS; W10.8XXAM; W10.8XXD; W10.8XXS; W10.9XXA; W10.9XXD; W10.9XXS; W11.XXXA; W11.XXXD; W11.XXXS; W12.XXXA; W12.XXXD; W12.XXXS; W13.0XXA; W13.0XXD; W13.0XXS, W13.1XXA; W13.1XXD; W13.1XXS; W13.2XXA; W13.2XXD; W13.2XXS; W13.3XXA; W13.3XXD; W13.3XXS; W13.4XXA; W13.4XXD; W13.4XXS; W13.8XXA; W13.8XXD; W13.8XXS; W13.9XXA; W13.9XXD; W13.9XXS; W14.XXXA; W14.XXXD; W14.XXXS; W15.XXXA; W15.XXXD; W15.XXXS; W16.011A; W16.011D; W16.011S; W16.012A; W16.012D; W16.012S; W16.021A; W16.021D; W16.021S; W16.022A; W16.022D; W16.022S; W16.031A; W16.031D; W16.031S; W16.032A; W16.032D; W16.032S; W16.111A; W16.111D; W16.111S; W16.112A; W16.112D; W16.112S; W16.121A; W16.121D; W16.121S; W16.122A; W16.122D; W16.122S; W16.131A; W16.131D; W16.131S; W16.132A; W16.132D; W16.132S; W16.211A; W16.211D; W16.211S; W16.212A; W16.212D; W16.212S; W16.221A; W16.221D; W16.221S; W16.222A; W16.222D; W16.222S; W16.311A; W16.311D; W16.311S; W16.312A; W16.312D; W16.312S; W16.321A; W16.321D; W16.321S; W16.322A; W16.322D; W16.322S; W16.331A; W16.331D; W16.331S; W16.332A; W16.332D; W16.332S; W16.41XA; W16.41XD; W16.41XS; W16.42XA; W16.42XD; W16.42XS; W16.511A; W16.511D; W16.511S; W16.512A; W16.512D; W16.512S; W16.521A; W16.521D; W16.521S; W16.522A; W16.522D; W16.522S; W16.531A; W16.531D; W16.531S; W16.532A; W16.532D; W16.532S; W16.611A; W16.611D; W16.611S; W16.612A; W16.612D; W16.612S; W16.621A; W16.621D; W16.621S; W16.622A; W16.622D; W16.622S; W16.711A; W16.711D; W16.711S; W16.712A; W16.712D; W16.712S; W16.721A; W16.721D; W16.721S; W16.722A; W16.722D; W16.722S; W16.811A; W16.811D; W16.811S; W16.812A; W16.812D; W16.812S; W16.821A; W16.821D; W16.821S; W16.822A; W16.822D; W16.822S; W16.831A; W16.831D; W16.831S; W16.832A; W16.832D; W16.832S; W16.91XA; W16.91XD; W16.91XS; W16.92XA; W16.92XD; W16.92XS; W17.0XXA; W17.0XXD; W17.0XXS; W17.1XXA; W17.1XXD; W17.1XXS; W17.2XXA; W17.2XXD; W17.2XXS; W17.3XXA; W17.3XXD; W17.3XXS; W17.4XXA; W17.4XXD; W17.4XXS; W17.81XA; W17.81XD; W17.81XS; W17.82XA; W17.82XD; W17.82XS; W17.89XA; W17.89XD; W17.89XS; W18.00XA; W18.00XD; W18.00XS; W18.01XA; W18.01XD; W18.01XS; W18.02XA; W18.02XD; W18.02XS; W18.09XA; W18.09XD; W18.09XS; W18.11XA; W18.11XD; W18.11XS; W18.12XA; W18.12XD; W18.12XS; W18.2XXA; W18.2XXD; W18.2XXS; W18.30XA; W18.30XD; W18.30XS; W18.31XA; W18.31XD; W18.31XS; W18.39XA; W18.39XD; W18.39XS; W18.40XA; W18.40XD; W18.40XS; W18.41XA; W18.41XD; W18.41XS; W18.42XA; W18.42XD; W18.42XS; W18.43XA; W18.43XD; W18.43XS; W18.49XA; W18.49XD; W18.49XS; W19.XXXA; W19.XXXD; W19.XXXS; R29.6; Z91.81

ICD-9: E804; E804.0; E804.1; E804.2; E804.3; E804.8; E804.9; E833; E833.0; E833.1; E833.2; E833.3; E833.4; E833.5; E833.6; E833.7; E833.8; E833.9; E834; E834.0; E834.1; E834.2; E834.3; E834.4; E834.5; E834.6; E834.7; E834.8; E834.9; E835; E835.0; E835.1; E835.2; E835.3; E835.4; E835.5; E835.6; E835.7; E835.8; E835.9; E843; E843.0; E843.1; E843.2, E843.3; E843.4; E843.5; E843.6; E843.7; E843.8¸E843.9; E880; E880.0; E880.1; E880.9; E881; E881.0; E881.1; E882; E883; E883.0; E883.1; E883.2; E883.9; E884; E884.0; E884.1; E884.2; E884.3; E884.4; E884.5; E884.6; E884.9; E885; E885.0; E885.1; E885.2; E885.3; E885.4; E885.9; E886; E886.0; E886.9; E887; E888; E888.0; E888.1; E888.8; E888.9; E917.5; E917.6; E917.7; E917.8; E929.3; E987; E987.0; E987.1; E987.2; E987.9; V15.88

HICDA: 14887110; 14887111; 07886215

Berkson Code: 125991
